# Effects of object motion on visual acuity in honeybees

**DOI:** 10.1242/jeb.251776

**Published:** 2026-04-22

**Authors:** Rishabh Desai, Matthew A. Garratt, Mandyam V. Srinivasan, Sridhar Ravi

**Affiliations:** ^1^ School of Engineering and Technology, University of New South Wales - Canberra, Campbell, ACT 2612, Australia; ^2^ Queensland Brain Institute, University of Queensland, St Lucia, QLD 4067, Australia

**Keywords:** *Apis mellifera*, Behaviour, Moving stimuli

## Abstract

Object motion is a fundamental visual cue for many animals, yet its role in modulating visual perception is not fully understood. The majority of previous studies have used stationary stimuli to investigate the visual acuity of insects such as honeybees. Here, we used a behavioural assay to investigate whether object motion influences the known bounds of bees' visual acuity. Using a Y-maze, we conducted binary choice experiments where honeybees (*Apis mellifera*) were trained to a stimulus and then tested with pairs of stimuli varying in shape (disc versus diamond), size and motion (stationary versus moving). When stimuli were stationary, bees perceived the change in shape only for targets that subtended visual angles >1.9 deg. However, when presented with a stationary and a moving stimulus, they consistently preferred the moving stimulus, even when both stimuli subtended angles as small as 0.44 deg. Furthermore, motion cues were found to override a learned preference for a familiar stationary shape. Most notably, bees successfully discriminated shapes at the smallest tested size of 0.44 deg when both targets were in motion, but not when they were stationary. These findings provide behavioural evidence that object motion can be a salient cue that not only drives choice but also enhances visual acuity in honeybees beyond previously established limits. This suggests that motion may facilitate the perception of fine spatial details and underscores the importance of incorporating dynamic cues when studying the sensory limits of animal vision.

## Teaser

**Summary:** Honeybees see better when flowers move. Behavioral experiments show that target movement could sharpen visual acuity. When foraging, bees also display strong preferences to moving targets.

## INTRODUCTION

Honeybees, like other central place foragers, live in actively changing environments. In their daily routine, they frequently encounter a range of moving objects such as local flora (flowers, grass, tree leaves and branches), airborne debris, conspecifics, looming stimuli such as predators, and other background noise. The motion of features in the environment is generated by numerous sources, including changing wind conditions, animal movement and rain (Peters et al., 2008). Within this dynamic scene they must parse object motion from self-generated optic flow (Srinivasan, 2021), segment objects from backgrounds (Ibarra et al., 2000; Werner et al., 2016), rapidly redirect attention to some selected targets (Paulk et al., 2014) and interact with them (Traniello et al., 2022; Zhang et al., 2004), all while integrating this information with other sensory pathways. Despite these challenges, bees remain remarkably adept at navigating through visual clutter (Jeschke et al., 2025), finding and evaluating flowers (Howard et al., 2021, 2019), and avoiding collisions (Singh et al., 2024), implying that motion is not merely tolerated but exploited as a salient cue. It is therefore plausible that motion plays a substantial role in their visual processing and could be a vital cue that informs their behaviour.

Underlying all these behavioural processes is the ability of insects to visually identify and process objects by resolving fine edges, shapes and sizes, both spatially and temporally across in their visual field (Srinivasan and Lehrer, 1985), which is defined by their visual acuity. In insects, visual acuity is fundamentally determined by the structure of the compound eye, primarily the interommatidial angle (Δφ) – the angle between the optical axes of adjacent facets – and the angular extents of the visual fields of individual ommatidia (the acceptance angle, Δρ) (Snyder, 1979). These physical limits result in a wide range of visual capabilities across different species, with interommatidial angles ranging from tens of degrees in primitive forms to ∼0.24 deg in dragonflies (Lancer et al., 2020; Sherk, 1978) and typically falling within a range of 1–3 deg (Land, 1997) in most fast-flying generalists such as bees (Dyer et al., 2008; Macuda et al., 2001), flies (Lawson and Srinivasan, 2020) and butterflies (Wright et al., 2023). The highest spatial resolution is typically found in predatory insects such as dragonflies and mantids, which possess specialized high-acuity regions, or ‘acute zones’, for tracking prey on the wing (Land, 1997).

Historically, behavioural (Giurfa and Vorobyev, 1998; Giurfa et al., 1996) and physiological (Labhart, 1980; Laughlin and Horridge, 1971) experiments investigating visual acuity in honeybees have complemented and built on each other to support or refine previous hypotheses. Early estimates projected the spatial acuity of honeybees to be around 3 deg (Giurfa and Vorobyev, 1998; Giurfa et al., 1996; Lehrer and Bischof, 1995). More recently, Rigosi et al. (2017) focused on physiological experimentation in evaluating the limits of spatial perception in bees. Their work in light-adapted conditions shows that detectability extends to objects as small as 0.6 deg. Between the photoreceptor limits noted above, and the eventual behavioural response in honeybees, a significant series of perceptual and cognitive processes occur (Paulk et al., 2009). Nearly all these studies have focused on evaluating visual acuity when the target is stationary.

A growing body of work hints that motion of an object can improve what a visual system can extract about its spatial detail, under the right conditions. The classical studies by Srinivasan and Bernard (1975) observed that, at high contrast and within a band of biologically plausible angular velocities, photoreceptor dynamics could become rate-sensitive, sharpening edge transients so that two close features are more separable than when static. Thus, motion can raise effective spatial resolvability rather than blur it. A recent study by Pang et al. (2025) also showed that recurrent feedback from the L1 and L2 visual interneurons in *Drosophila* enhances the perceived contrast of moving edges, making moving objects easier to detect than static ones. Second-order motion characteristics such as contrast changes can also be exploited in animals such as praying mantises to detect moving targets, demonstrating enhanced sensitivity to object motion (Nityananda et al., 2019). Similar studies in humans show that neural aftereffects of brief motion adaptation can produce acuity improvements, possibly by shifting sensitivity toward higher spatial-frequency channels, suggesting that motion signals can retune spatial perception (Tagoh et al., 2022). However, it should be noted that the sensitivity enhancements noted by Nityananda et al. (2019) and Tagoh et al. (2022) are likely to be driven by attentional mechanisms, and may not directly translate to photoreceptor-level dynamics. Evidence also suggests the improvement of effective acuity in resolving fine spatial details, where motion can enhance spatial acuity through a mechanism that integrates information over time to reconstruct a more detailed image (Patrick et al., 2017).

From a behavioural context, previous studies have noted preferential selection of moving flowers, rather than their stationary counterparts (Desai et al., 2024). A proposed reason for this was the additional visual salience of a moving flower. It is possible that targets moving with ecologically relevant profiles can be detected easier and earlier, leading to a ‘see first, choose first’ strategy for foraging decisions in honeybees. It is hypothesised that object motion can augment target detection and, consequently, inform the process of decision-making in honeybees. Among the wealth of visual cues that modulate this process, object motion could facilitate the detection and characterisation of features in the environment. Thus, a behavioural exploration of visual acuity in the context of dynamic stimuli, as presented here, could enrich the current understanding of the processes underlying flower detection by investigating more explicitly how visual cues translate into behavioural decisions.

In this study, we used the well-established Y-maze behavioural assay to evaluate the effect of stimuli motion on the ability of honeybees to differentiate between visual stimuli. Established thresholds of spatial acuity from literature were used and compared with experiments where visual targets were augmented with motion. By giving bees binary choices, the modulation of their object detection and visual acuity was studied.

## MATERIALS AND METHODS

The experiments were conducted during the winter of 2024 at the University of New South Wales in Canberra, where colonies of western honeybees (*Apis mellifera* Linnaeus 1758) were maintained on campus*.* A gravity feeder (feeder A) containing warm 100% v/v sugar solution was used to attract foraging honeybees to the experiment site. Once a healthy population of approximately 30 bees continued to visit the feeder even when the sugar concentrations were dropped to as low as 10% v/v, a subset of the bees was recruited and trained to visit the Y-maze used for the experiments. Within the Y-maze there were two identical disc-shaped objects located on either side, presenting nectaries with sugar solution. During the training phases, honeybees were allowed to forage on the discs throughout the day. During the experimental stages, however, the training discs were replaced by test stimuli in which the nectaries were kept empty.

### Experimental maze

**Fig. 1.**
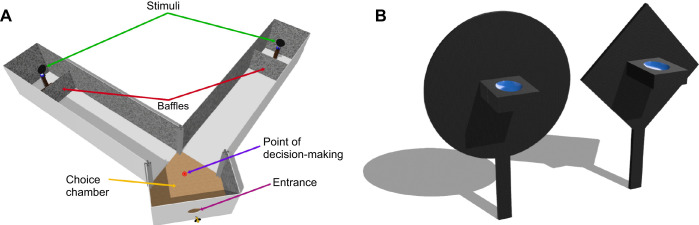
**A schematic diagram of the experimental setup involving a Y-maze with a central decision chamber for binary choice experiments highlighted below.** (A) An isometric view of the Y-maze. (B) Isometric representations of the disc-shaped (left) and diamond-shaped (right) floral stimuli used in the experiments, with a feeder at the centre of each stimulus. Blue colour in the feeder represents sugar solution.

All the experiments in this study investigated choice behaviour in honeybees. The experiments explored discrimination of very fine differences in stimulus size and movement. As a result, it was important to further isolate any impact on their decision-making arising from the physical proximity of choice stimuli. A Y-shaped maze was designed and deployed for conducting these experiments, as outlined in Fig. 1A. Several previous studies have used Y-shaped mazes for conducting binary choice experiments in bees (Ikeno, 2004; Nouvian and Galizia, 2019; Srinivasan and Lehrer, 1988).

The maze consisted of three parts. The bees first entered a central chamber 205 mm long, which served as the decision-making area. From the centre of this chamber, the bees could view two stimuli (defined by their centres) that were separated by 90 deg, which is within the bees' frontal visual field, where pattern recognition and spatial perception is strongest (Giger and Srinivasan, 1997; Lehrer, 1990; Wehner, 1972). After making their decision, the bees would fly through either of the two identical 1300 mm long arms of the maze, at the end of which they would land on the stimulus in the chosen arm. Each stimulus consisted of a disc mounted on top of a pedestal connected to a servo motor. Baffles (200 mm high) were used to hide the ancillary components and ensure that only the stimuli were visible to the bees. To enhance spatial perception, the internal walls of the maze and the baffles were covered with a 1/f noise pattern as described in previous studies (Desai et al., 2024; Ravi et al., 2019, 2020). The maze was covered with transparent UV-blocking acrylic glass, so that the observer had a clear view of the bees' behaviour and their choices.

### Stimuli

All the experiments in the series consisted of two phases, as in previous experiments: a training phase where bees were acclimatised to the maze, and a testing phase where their choices were recorded (Desai et al., 2024). Two different types of stimuli were used for the experiments: a diamond shaped floral body as described in Fig. 1B (right) and a disc shaped floral body as described in Fig. 1B (left).

Apart from the shape, the two stimuli were identical in every other respect. They were 3D-printed from a single spool of PETG filament to maintain identical colour, texture and reflective properties. Each of these two types of stimuli were printed in four different sizes. Defined as diametric lengths for the discs and diagonal lengths for the diamonds, the sizes were 10, 20, 43 and 70 mm. The angular size of each stimulus was calculated from the perspective of a central decision-making point in the choice chamber (see Fig. 1A). This was computed by determining the angle subtended between two imaginary lines drawn from this point to the stimulus's left and right edges. Using this method, the four physical sizes corresponded to angular sizes of 0.44, 0.88, 1.9 and 3.1 deg.

The stimulus sizes chosen in this study were based on the best-known measures of spatial acuity in honeybees. Angular sizes were chosen such that the largest size was slightly larger than 3 deg, and the smallest size was slightly smaller than 0.6 deg. For the training phases, honeybees foraged only on the largest sized flowers.

Each flower moved in an arc-like side-to-side motion. The amplitude and frequency of flower oscillation were chosen as ±20 deg and 3.5 Hz, respectively. This combination was chosen such that the bees could observe multiple oscillations of the flower during their flight through the tunnel. Furthermore, based on trial and error, this movement profile was noted to cause minimal disruptions to the bees during landing, as observed in previous experiments (Hennessy et al., 2020). The movement was actuated by servo motors mounted on raised wooden pedestals. The flowers were carried by stems which were moved using servos controlled by Arduino Nano microcontrollers. The servos were powered by external 6 V Ni-Cd batteries.

Each flower also contained a concave nectary (a feeder) that bore sugar solution to motivate the bees to visit the flowers regularly. However, during the testing phase, while nectaries were still present on each flower to maintain consistency with the training flowers, neither stimulus bore sugar solution. Moreover, the flowers used in training were isolated and never used in any of the tests, to eliminate the influence of olfactory cues arising from foraging.

### Training and experiments

Before initiating the tests, the bees were trained to enter the maze, where two identical stationary training stimuli representing flowers were placed on each of the two ends of the Y-maze. The size of the training flowers was always 100 mm, irrespective of their shape. Honeybees could choose either of the two flowers, and previous experiments (Desai et al., 2024) indicate that for identical flowers, a control population in the training phases would be equally likely to choose either flower, with no statistically significant difference between the two choices. The population of visiting bees grew gradually with time, and testing was commenced when 10–15 bees were frequenting the training flowers.

A total of four different experiments were conducted. Each experiment was conducted in bouts of 10 bees, and after each bout, bees were temporarily encapsulated to avoid repeated visits from the same bees. In total, five sets of 10 honeybees were tested for each experiment. After the end of each test, training was briefly resumed until a new population of bees, sufficient for the next test, was recruited.

During the testing procedure, the training flowers were first replaced by a different set of test flowers, with sizes varying according to the testing requirements. None of the test flowers contained sugar solution. Honeybees were let in, one at a time, by controlling their entry through a sliding panel at the entrance of the maze that acted like a door and defaulted to a closed position, unless opened. The bees, once let in, would make a choice in the decision chamber, and move toward their choice of flower stimulus. Because the two flowers were located in different arms of the Y-maze, it was practically impossible for a bee to turn toward the other flower after making its initial choice. When a bee had chosen to enter one arm of the Y-maze, it was not observed to return to the central chamber and enter the other arm. Choices were recorded based on the flower on which the bee landed. Once a bee had reached the end of the maze, it was extracted using a nonlethal bug vacuum (Bug-Vac, BioQuip, Rancho Domingues, CA, USA) and placed in a storage container provisioned with cotton balls soaked in sugar solution for its sustenance.

During testing, standard practices for cleaning and stimulus side swapping were followed, as in other experiments, to avoid the effects of side biases or olfactory influences on the bees' decision making. Stimuli were swapped across the arms from time to time in a pseudo-random manner during each set, and after every 2–3 landings the nectary was wiped clean with 100% ethanol followed by tap water.

Because multiple experiments were conducted over several days, bees captured during the experiments were released at the end of the day before dusk, after being marked on the abdomen with a Posca paint marker to ensure that any marked bee participating in subsequent experiments was eliminated from the records. Any marked bees attempting to return were excluded from the maze or their data discarded. The colony maintained a sufficient foraging population to provide naive subjects throughout the study period. No significant depletion of the forager pool was observed.

#### Experiment 1

The first experiment was performed to establish a baseline of the spatial acuity of honeybees in stationary conditions by testing shape discrimination abilities under varying stimulus sizes.

**Fig. 2.**
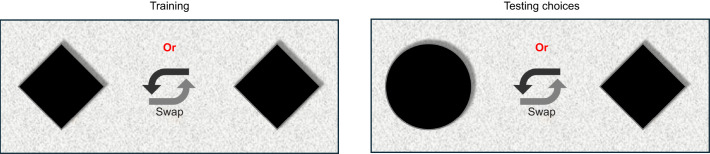
**A view of the stimuli for experiment 1, as observed by honeybees upon entering the maze.** Bees were trained with stationary diamonds and then offered a simultaneous choice between a stationary diamond and disc.

For this, bees were first trained on two identical diamond-shaped flowers of the largest size (3.1 deg). After training, experiments were conducted using four different stimulus sizes: 0.44, 0.88, 1.9 and 3.1 deg. For each of these sizes, experiments were conducted with *N*=50 bees, in 5 sets of 10 bees each. In the tests, honeybees were given a choice between a stationary diamond-shaped flower and a stationary disc-shaped flower (hereafter referred to as a diamond and a disc) (see Fig. 2). It was postulated that the bees would be likely to choose the diamond over the disc; therefore, for the data collection, the position of the diamond in each trial and the subsequent side that the honeybee chose were noted. The choice was scored as a ‘1’ if the honeybee chose the arm containing the stationary diamond, and ‘0’ if it chose the arm containing the stationary disc. Throughout the experiments, the positions of the two test stimuli were swapped at random to avoid any influence of possible side biases. This was repeated for each flower size, to examine whether there is a size threshold below which shape discrimination was not possible.

#### Experiment 2

**Fig. 3.**
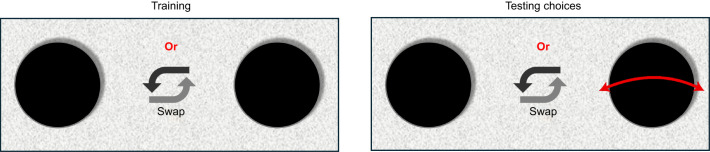
**A view for the design of experiment 2, where honeybees underwent training on stationary discs followed by a binary choice between identical moving and stationary discs.** Stimulus sizes were varied in a descending order.

This experiment sought to identify the size threshold where bees could perceive differences in movement of the flower. In this experiment, the honeybees were trained on two identical stationary discs. After the training, they were given a choice between two identical discs, one stationary and the other moving (see Fig. 3). Four different stimulus sizes were used, as in experiment 1. Each test involved *N*=50 bees and only one trial was recorded per bee. A total of 50 (number of bees)×4 (stimulus sizes) individuals were included in the experiment. It was hypothesised that differences in flower movement would be discernible to honeybees at sizes <1.9 deg, the threshold where bees failed to perceive differences between flower shapes. Therefore, in the tests, the position of the moving disc in each trial was noted along with the choice made by each honeybee. When the bee chose the moving disc, the choice was scored as a ‘1’; otherwise, it was ‘0’. The tests were first conducted for the largest size and then repeated for decreasing flower sizes. For each set of tests, the flower positions during testing were swapped at random. The goal was to understand how the results vary when a difference in flower movement (moving or not moving) is introduced as a visual cue in experiment 1 instead of shape differences.

#### Experiment 3

**Fig. 4.**
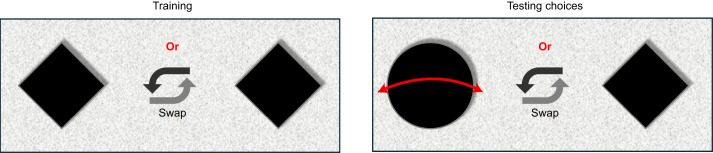
Experimental design for experiment 3, where honeybees were trained on stationary diamonds followed by a test which offered a choice between a stationary diamond and a moving disc.

Following experiment 1, which investigated shape discrimination, and experiment 2, which investigated movement-based discrimination, this experiment was a follow-up to investigate decision-making under the simultaneous presence of both visual cues by placing them in competition with each other and determining whether one had a larger impact than the other. For this experiment, honeybees were trained on a stationary diamond. Later, for tests, the bees were required to choose between a stationary diamond (like the one used during training) and a moving disc (see Fig. 4). For this part of the study, only the largest flower size (i.e. 3.1 deg) and the smallest size (i.e. 0.44 deg) were used, resulting in two different tests each involving a population of 50 individuals. Flower positions were swapped between the two sides of the maze at random within each test set. It was hypothesised that existing preferences arising from shape familiarity would supersede preference for a moving flower with an unfamiliar shape. When the bees chose the moving disc, the choice was scored as ‘1’; otherwise, it was scored as ‘0’.

#### Experiment 4

**Fig. 5.**
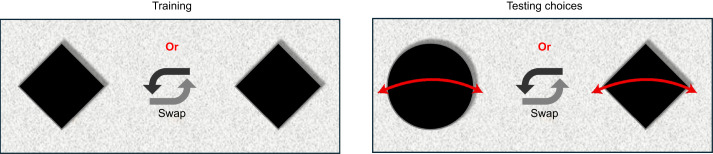
Experimental design for experiment 4, where honeybees were trained on a stationary diamond and then offered a choice between a moving disc and a moving diamond.

This experiment, in contrast to experiment 3, was conducted to investigate whether shape discrimination of small objects is enhanced by the presence of motion. For this experiment, the bees were trained on identical stationary diamonds placed on either side of the maze, followed by a test condition where they were given a choice between a moving disc and a moving diamond (see Fig. 5). The position of the moving diamond for each trial was noted and compared with the subsequent honeybee choice. In the tests, the choices were scored a ‘1’ when the choice was for the moving diamond, and ‘0’ when it was for the moving disc. This experiment involved only the smallest flower size (0.44 deg) and the largest flower size (3.1 deg), as in experiment 3. As in the other experiments, the positions of the test stimuli positions were swapped at random.

### Statistical analysis and plotting

The data recorded for the experiments were tabulated in Microsoft Excel (ver. 2502). Analysis was done using the R programming language (version 4.3.3) in RStudio version 2024.04.2+764 (RStudio, PBC, Boston, MA, USA). As each set of data recorded contained binary choices, statistical analysis was performed using chi-squared tests for pairwise comparisons with Yate's continuity correction and a two-tailed binomial test to compare the choices against a chance-based normal distribution of 50% (expected value=0.5; α=0.05). Plotting was done using MATLAB 2024b version 3 (MathWorks, Natick, MA, USA). Figure design and editing were done with Microsoft PowerPoint ver. 2501 and GIMP 2.10.36, respectively.

### Ethical notes

In line with common scientific practice and regulations, formal ethical approval is not required for behavioural studies involving invertebrates such as honeybees (*A. mellifera*). Nevertheless, all procedures were designed and conducted with the utmost consideration for their welfare. The use of sugar solution as a reward provided a source of nourishment that mimics their natural foraging. Following each trial, bees were gently collected using a non-lethal bug vacuum and temporarily placed in a holding container. This container was well ventilated and provisioned with cotton soaked in sugar solution to provide sustenance and a safe environment. All bees were released unharmed at the end of each day's experiments, after which they were able to continue their lifestyle of foraging in a natural outdoor environment.

## RESULTS

### Experiment 1: Spatial acuity thresholds for shape detection

**Fig. 6.**
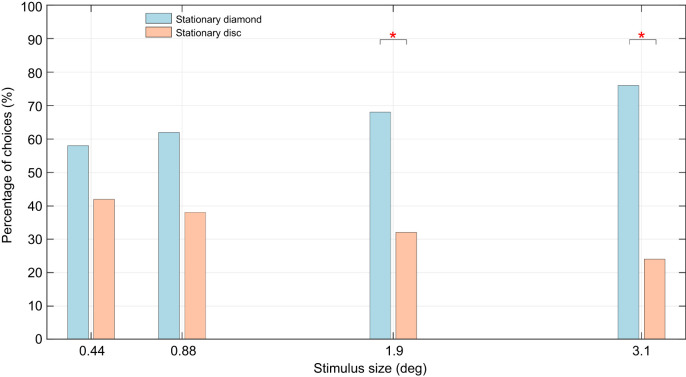
**Results from experiment 1 show that honeybees could discriminate shapes for the larger (3.1 and 1.9** **deg) flowers but failed to do so for the smaller sizes (0.88 and 0.44** **deg).** Asterisks indicate significant differences (*P<0.05) between the bees' choices for the two shapes.

In experiment 1, when bees were trained on the stationary diamonds and then tested by presenting them with a choice between a stationary disc and a stationary diamond of the same size, they showed a significant preference for the stationary diamond. For the 3.1 deg flowers, 76% of the landings were on the diamond, which was markedly higher than the landings on the disc (*P*=0.0007, *N*=50, chi-squared test, ϕ=0.482; Fig. 6; Table S1). A similar result was obtained when the bees were tested on flower sizes of 1.9 deg: 68% of the landings were on the diamond, which was significantly higher than the landings on the disc (*P*=0.0232, *N*=50, ϕ=0.321; Fig. 6; Table S1). However, there was no significant difference between the choices for the two flower shapes for the smaller flower sizes of 0.88 deg (*P*=0.1491, *N*=50, ϕ=0.204) and 0.44 deg (*P*=0.3818, *N*=50, ϕ=0.124; Fig. 6; Table S1).

### Experiment 2: Motion detection persists where shape discrimination fails

**Fig. 7.**
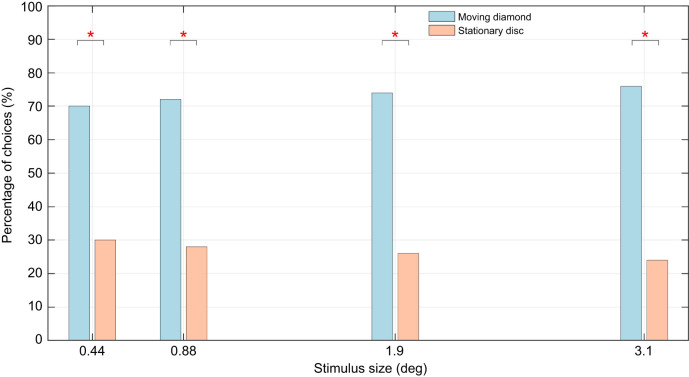
**Results from experiment 2 indicate that motion detection persists even for the smaller flower sizes.** Moving discs attracted significantly more landings than stationary discs. Asterisks indicate significant differences (*P<0.05) between the bees' choices.

In experiment 2, the bees were trained on two identical stationary discs, and subsequently tested by presenting them with a choice between a stationary and a moving disc. The experiment was carried out for four different disc sizes: 3.1, 1.9, 0.88 and 0.44 deg. In the tests, the bees showed a strong preference for the moving disc for all four sizes: 75%, 74%, 72% and 70% for the sizes 3.1, 1.9, 0.88 and 0.44 deg, respectively. The preferences were statistically significant in all four cases: 3.1 deg (*P*=0.007, *N*=50, ϕ=0.48), 1.9 deg (*P*=0.0018, *N*=50, ϕ=0.44), 0.88 deg (*P*=0.0047, *N*=50, ϕ=0.4) and 0.44 deg (*P*=0.0109, *N*=50, ϕ=0.36; Fig. 7; Table S1).

### Experiment 3: Shape versus motion – decisions under competing visual cues

**Fig. 8.**
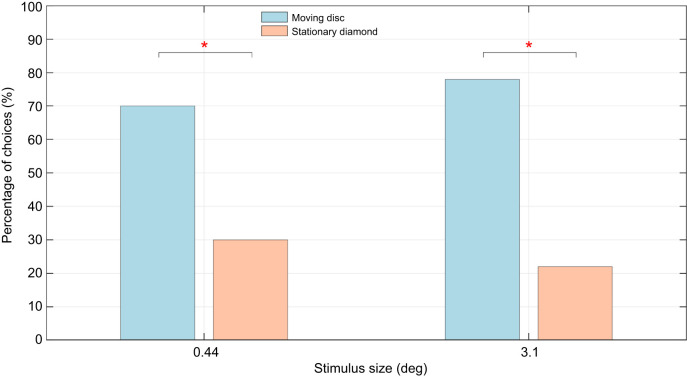
**Results of experiment 3 reveal a significant priority given to flower motion in honeybee landing decisions in the presence of shape differences.** When given a choice between familiar stationary shapes and unfamiliar moving shapes, bees showed a significantly stronger preference for the moving shape, for the largest and the smallest sizes. Asterisks indicate significant differences (*P<0.05) between the bees' choices.

The results of experiments 1 and 2 highlight a difference in flower discrimination ability between shape-based and motion-based choices, where the bees were able to distinguish motion differences in flowers at sizes where shape discrimination in stationary flowers was not possible. However, experiment 3 addresses the question whether honeybees process motion and shape cues independently of one another, and whether motion cues can override a learned shape cue. The smallest and the largest sized flowers (0.44 and 3.1 deg) were used in these experiments. It was observed that when given a choice between a stationary diamond identical to the one on which they were previously trained, and a moving disc of the same size, the disc took precedence. For the 0.44 deg size, the landing choices for the moving disc were markedly higher: at 70% compared with 30% for the stationary diamond. The choice frequencies were significantly different (*P*=0.0103, *N*=50, ϕ=0.363; Fig. 8; Table S1). Similar results were obtained for the largest flower size of 3.1 deg: the preference for the moving disc was even stronger (78%), and more significant (*P*=0.0002, *N*=50, ϕ=0.52; Fig. 8; Table S1).

### Experiment 4: Flower motion enhances shape discrimination

**Fig. 9.**
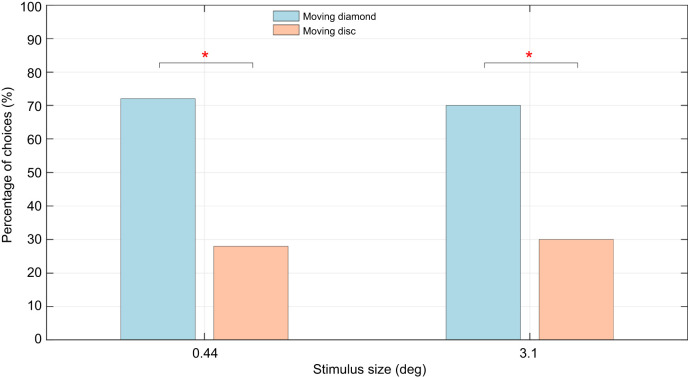
**Results of experiment 4 reveal a significant preference for the moving diamonds.** When both stimuli were in motion, bees showed a significantly stronger preference for the diamond, matching the shape on which they were trained. Asterisks indicate significant differences (*P<0.05) between the bees' choices.

Experiment 4 sought to assess the bees' preferences when motion was present across all stimuli. Only the largest and the smallest sizes (3.1 and 0.44 deg) were used in this experiment. Experiment 4 was like experiment 1, in that it investigated shape discrimination, but in the context of movement. Experiment 1 revealed that the two shapes (disc and diamond) could be discriminated only at the two larger sizes (3.1 and 1.9 deg). However, in experiment 4, the bees were able discriminate at the largest size (3.1 deg) as well as the smallest size (0.44 deg). The bees' preference for the trained shape (diamond) was 70% for the 3.1 deg flower (*P*=0.0103, *N*=50, ϕ=0.363) and 72% for the 0.44 deg flower (*P*=0.0027, *N*=50, ϕ=0.486; Fig. 9; Table S1).

## DISCUSSION

### Detection limits under static conditions

In this study, the effect of stimulus movement on choice preferences of honeybees was investigated through a series of experiments. A central hypothesis of this study is that object motion influences behaviour and landing decisions in honeybees. The crux of the hypothesis centres around the question of whether object motion, especially within the context of flower motion, can contribute to the existing knowledgebase of their visual acuity. Earlier behavioural studies (Giurfa and Vorobyev, 1998; Giurfa et al., 1996; Lehrer and Bischof, 1995; Srinivasan and Lehrer, 1988) have suggested an angular resolution of no better that 3 deg, accounting for the influences of chromatic and achromatic variations. Physiological studies, in contrast, previously claimed a marginally lower threshold for detectability in dark-adapted scenarios of approximately 2.6 deg (Labhart, 1980; Laughlin and Horridge, 1971). However, recent work by Rigosi et al. (2017) reported a much finer ability of bees to discriminate objects, and suggested minimum detectable target sizes as low as 0.59 deg in the frontal visual field.

The goal of this study was to determine, from a behavioural context, whether target motion can affect visual perception. The first experiment, where four stimulus sizes (0.44, 0.88, 1.9 and 3.1 deg) were used, revealed the inability of bees to perceive the differences between two different shapes when the stimulus size was <1.9 deg (see Figs 2 and 6). These results nominally align with some previous observations (Horridge, 2005) while also contrasting with other similar behavioural studies (Greiner et al., 2004; Somanathan et al., 2009) that have shown the ability of bees to detect changes in features of even smaller targets. A direct comparison of the limits observed here with those reported in other studies is difficult because of several differences in the experimental methodologies that were employed. Factors such as illumination, and contrast of the target against the background, could affect detection and discrimination capacities, and thus affect the bees' choices. The goal of experiment 1 was to ascertain a more subjective baseline of the behavioural limitations of the bees' angular sensitivity in the frontal eye region. The results of experiment 1 serve as a control for comparison against the other experiments.

### Preference for moving over stationary stimuli

The results from experiment 2 reveal that motion significantly modulates stimulus choices during foraging. When presented with a choice between a moving and a stationary stimulus that were otherwise identical, bees tended to choose the moving stimulus even when it was as small as 0.44 deg (see Figs 3 and 7). The size at which bees were able to distinguish between the stimuli, as a result of motion of one of them, is significantly smaller than that observed in the static conditions examined in experiment 1. The lower limit of stimulus size is also lower than the lowest previously known thresholds of approximately 0.59 deg observed in Rigosi et al. (2017). There are several substantial differences between their study and the present one, which could provide further context to the differing results. In Rigosi et al. (2017), bees were presented with a virtual stimulus moving at a constant horizontal velocity of 40 deg s^−1^ in a left-to-right motion. The moving stimulus used in the present study has considerably higher peak angular velocities (as high as 500 deg s^−1^) and average velocities of around 350 deg s^−1^. Additionally, the physical stimuli used in the present study present three-dimensional depth information as well as texture and lighting-based differences, typical of real environments. The sinusoidally oscillating flowers also present temporal variations in angular velocity and acceleration. A potential reason that spatial acuity thresholds reported here are lower than those from previous studies could be the use of such oscillating motion, which is more representative of natural flower movements such as those triggered by wind or animal disturbances, and could provide additional spatial and temporal information. It should also be noted that the honeybees tested in Rigosi et al. (2017)’s study were tethered, as opposed to the free-flying honeybees in our study.

Studies in many other insects have previously observed similar capacities for the identification of small visual features. Among flying insects, dragonflies, with Δφ of approximately 0.24 deg, demonstrate some of the smallest recorded thresholds (Lancer et al., 2020; Sherk, 1978). Insects with Δφ less than 1 deg are more commonly involved in predation and chasing (Land, 2009; Tarawneh et al., 2018). Although predation and chasing are not as frequent in honeybees, goal seeking and target identification for detection and recognition of flowers remain an essential part of their daily activities. This would potentially demand the same robust detectability, as identified in this study and previous research with honeybees (Labhart, 1980; Laughlin and Horridge, 1971; Rigosi et al., 2017; Srinivasan and Lehrer, 1988).

### Hierarchical preferences

It is possible that honeybees prioritise motion cues over other cues such as object shape. In a previous study (Desai et al., 2024), bees were presented a choice between identical flowers that were a different shape than the one on which they had been trained, but with one of them in motion. Bees demonstrated a significant preference for the moving flower even when the two flowers had unfamiliar shapes. The tests conducted in experiment 3 here are an extension of this study, incorporating multiple contrasting and competing cues. During testing, bees were given a choice between a familiar shape (matching the training stimuli) that was stationary, or a new, unfamiliar shape that was moving. At a flower size of 3.1 deg (a size at which the bees have been shown to distinguish shapes), it would be expected that bees would be more likely to choose the familiar shaped flower. In contrast, for cases where the flowers were 0.44 deg in size, they would be expected to predominantly choose the moving disc owing to its increase salience as noted in experiment 2. However, we found that even in the presence of a familiar shape (at stimulus size of 3.1 deg), bees' choices were predominantly governed by the presence of flower motion, with a significant majority landing on the unfamiliar moving disc (experiment 3, see Fig. 8).

We are not suggesting that motion-based biases in decision-making would supersede the processing of other visual cues. There are indeed a large variety of additional visual features such as object colour (Giurfa et al., 1995; Romero-González et al., 2020), symmetry (Horridge, 1996), visual contrast (Hempel de Ibarra et al., 2014), the presence of outwards radiating floral features (Lehrer et al., 1997) and olfactory cues (Moreno et al., 2022) that can influence their decision-making. The scope of the present study does not allow a comprehensive determination of the relative importances of the various visual cues that play a role in the bees' final decision. Rather, the results from experiment 3 indicate that motion, by itself, could potentially be a source of visual salience. Owing to its inherently temporal nature as observed in the honeybees' natural environment, it cues honeybees earlier than some other visual characteristics of the flower. It is possible that, as bees eventually move closer, features such as colour and shape could play a more vital role, as bees begin to prioritise more distinctively floral properties.

Srinivasan and Bernard (1975) have previously proposed the enhancement of visual acuity in the presence of moving objects that move at biologically viable velocities under high contrast conditions. They posit this is due to the quicker photoreceptor response times under bright light adaptation and an increase in rate sensitivity, where they are more sensitive to changes in brightness rather than the absolute brightness. In experiment 3, bees are similarly presented with high contrast visual conditions, where they could observe large temporal changes owing to the flower motion, leading to additional visual salience, and thus, a preference for the moving stimulus.

In some other insects such as dragonflies, research has also investigated the responses of specialised small target motion detectors (STMDs) that are responsible for detecting and tracking small objects such as prey against complex backgrounds. Through a process of spatial facilitation, targets that move along a continuous, predictable trajectory will selectively ‘pop out’ from background noise (Nordström et al., 2011). Moreover, the CSTMD1 neuron described in that study can draw selective attention to such targets, when primed to a particular motion trajectory, even in the presence of other high-contrast distractors in the visual field. The preference of bees for the moving stimulus in experiment 3 similarly prioritises the powerful cue of continuous motion, effectively overriding the learned preference for the stationary shape.

### Motion enhancing visual acuity

The results from experiment 4 indicate that motion appears to enhance the limits of shape perception. Fundamentally, the tests conducted in experiment 4 are similar to those in experiment 1, except that all the stimuli are moving in the case of experiment 4. Yet, the results from the two experiments are markedly different. By itself, discrimination of stationary shapes was found to have larger size thresholds (Fig. 6). However, when motion was added, honeybees were able to differentiate shapes even at the smallest size (Fig. 9). The physiological reasons for this are unclear, as it may be argued that movement could also increase sensor noise because it introduces an additional variable to contend with, unlike the case with static stimuli. In contrast, dynamic fluctuations in the stimuli may serve to accentuate critical visual features – such as edges and contours – and potentially increase the visual contrast between shapes. Whereas motion appears to act as the primary cue for initial detection in experiment 3 (see Fig. 8), experiment 4 demonstrates that motion can also act as a facilitative cue. This contrast suggests that the role of motion might be context-dependent: motion can not only drive early attraction but can also enhance the perceptual clarity of shape details when the available cues are dynamic. Although such a suggestion is novel to hymenopterans, studies have observed similar behaviour in other organisms where temporal changes elicit attention-like processes. Nityananda (2016) discussed salience filters cued to preferential frequencies of sound and colours in insects. Attention-like processes dependent on visual flicker were also observed in fruit flies (Van Swinderen, 2012). Among mammals, mice were also found to be highly sensitive to temporal contrast (Umino et al., 2018). With growing evidence to suggest a role of temporal contrast for attention-like processes in all types of organism, this hypothesis could potentially be one such way in which honeybees employ object motion to enhance the perception of other visual cues.

Rigosi et al. (2017), in their experiments, identified a threshold of approximately 0.59 deg, which would closely represent the physiological limit of spatial acuity in honeybees. Because recordings were made from single photoreceptors in their experiments, the measured thresholds of acuity are likely to be limited by the size and acceptance angle of the photoreceptors themselves. In the present experiments, feature detection in moving flowers is observed at an angular size of 0.44 deg, which is approximately 25% lower. This raises an interesting dilemma: behaviourally, honeybees in the present study appear to demonstrate better visual acuity than what their photoreceptor physiology would allow for.

Although it is difficult to pinpoint the neural basis for the increased acuity, a potential explanation could be that processing at higher levels of the visual pathway could render resolution greater than the limits identified from the optics of a single photoreceptor. A previous study in *Drosophila* (Juusola et al., 2017) has demonstrated hyperacute vision beyond the optical limits of their compound eyes through mechanical contractions in photoreceptors resembling microsaccades, and refractory phototransduction. Although microsaccadic motion should render the objects in the visual field blurry, motion was found to bring enhanced resolution of objects by converting fine spatial details into sharp temporal signals.

Studies with bioinspired visual sensors have also observed similar enhancements in visual acuity when perceiving a moving image. Viollet and Franceschini (2010) demonstrated visual acuity as low as 0.04 deg, demonstrating a 70-fold improvement. Juston et al. (2014) also noted a similar marked increase in spatial resolution through microvibrations in the visual sensor when compared with static conditions. Srinivasan and Bernard (1975) observed in insects that the enhancement of visual acuity from object motion occurs over very specific biologically viable velocities. At velocities lower or higher than these, object detection could be negatively impacted. Even in dragonflies, detection by STMDs can be adversely affected when prey employ flight trajectories that keep retinal image direction nearly constant, making the moving prey look stationary to the observer – exploiting motion processing to defeat detection (Mizutani et al., 2003). Although these arguments centre around the temporal contrast from moving targets, it is also important to note that honeybees, like all other organisms, have limits of temporal resolution, and movement faster than their flicker-fusion frequency can effectively render the object ‘invisible’ (Srinivasan and Lehrer, 1984).

Although literature suggests that downstream neural processes could enhance the spatial resolution beyond the limits measured at individual photoreceptors, future studies need to dissect these processes to better understand the pathways triggered by object motion that enhance visual acuity and impact behaviour.

## Supplementary Material

10.1242/jexbio.251776_sup1 Supplementary information
