# Supplementary material for: Effects of object motion on visual acuity in honeybees
Source: J Exp Biol. 2026 Apr 22;229(8):jeb251776. doi: 10.1242/jeb.251776 (PMC13143216; doi:10.1242/jeb.251776)
Supplement: Supplementary information [file jexbio-229-251776-s1.pdf]

Table S1. A tabulated summary of the results of binomial analyses of the four experiments. P-values shown in red indicate insignificant results and those shown in green indicate statistically significant differences.

Test	Diametric Size (mm)	Angular Size (degrees)	Population (N =)	Chi-Squared Test		Binomial test	
				p-value	Phi	p-value	Cohen's h
Experiment 1: Purely shape-based Discrimination	10	0.44	50	0.3818	0.124	0.1611	0.161
	20	0.88	50	0.1491	0.204	0.1189	0.242
	43	1.9	50	0.0232	0.321	0.0153	0.368
	70	3.1	50	0.0007	0.482	0.0003	0.547
Experiment 2: Purely movement-based Discrimination	10	0.44	50	0.0109	0.36	0.0066	0.412
	20	0.88	50	0.0047	0.4	0.0026	0.456
	43	1.9	50	0.0018	0.44	0.0009	0.501
	70	3.1	50	0.0007	0.48	0.0003	0.547
Experiment 3: Shape & Movement as competing visual cues	10	0.44	50	0.0103	0.363	0.0066	0.412
	70	3.1	50	0.0002	0.52	0.0001	0.594
Experiment 4: Shape discrimination with both stimuli moving	10	0.44	50	0.0027	0.486	0.0026	0.456
	70	3.1	50	0.0103	0.363	0.0066	0.412

Journal of Experimental Biology • Supplementary information
